# Supplementary material for: Predicting visual function by interpreting a neuronal wiring diagram
Source: Nature. 2024 Oct 2;634(8032):113–23. doi: 10.1038/s41586-024-07953-5 (PMC11446822; doi:10.1038/s41586-024-07953-5)
Supplement: Supplementary file 6 — CRF and ERF predictions for individual TmY4 and TmY9 cells. Analogous to Supplementary Data 3, but for TmY target types. Shown are the top four monosynaptic pathways, the strongest pathway passing through each of the top ten intermediary types (ranking from Extended Data Fig. 7), and the trisynaptic pathway Tm1–TmY–Dm3–TmY (see the section entitled Prediction of spatial normalization). [file 41586_2024_7953_MOESM6_ESM.zip › DataS4/TmY9q/720575940629655802.pdf]

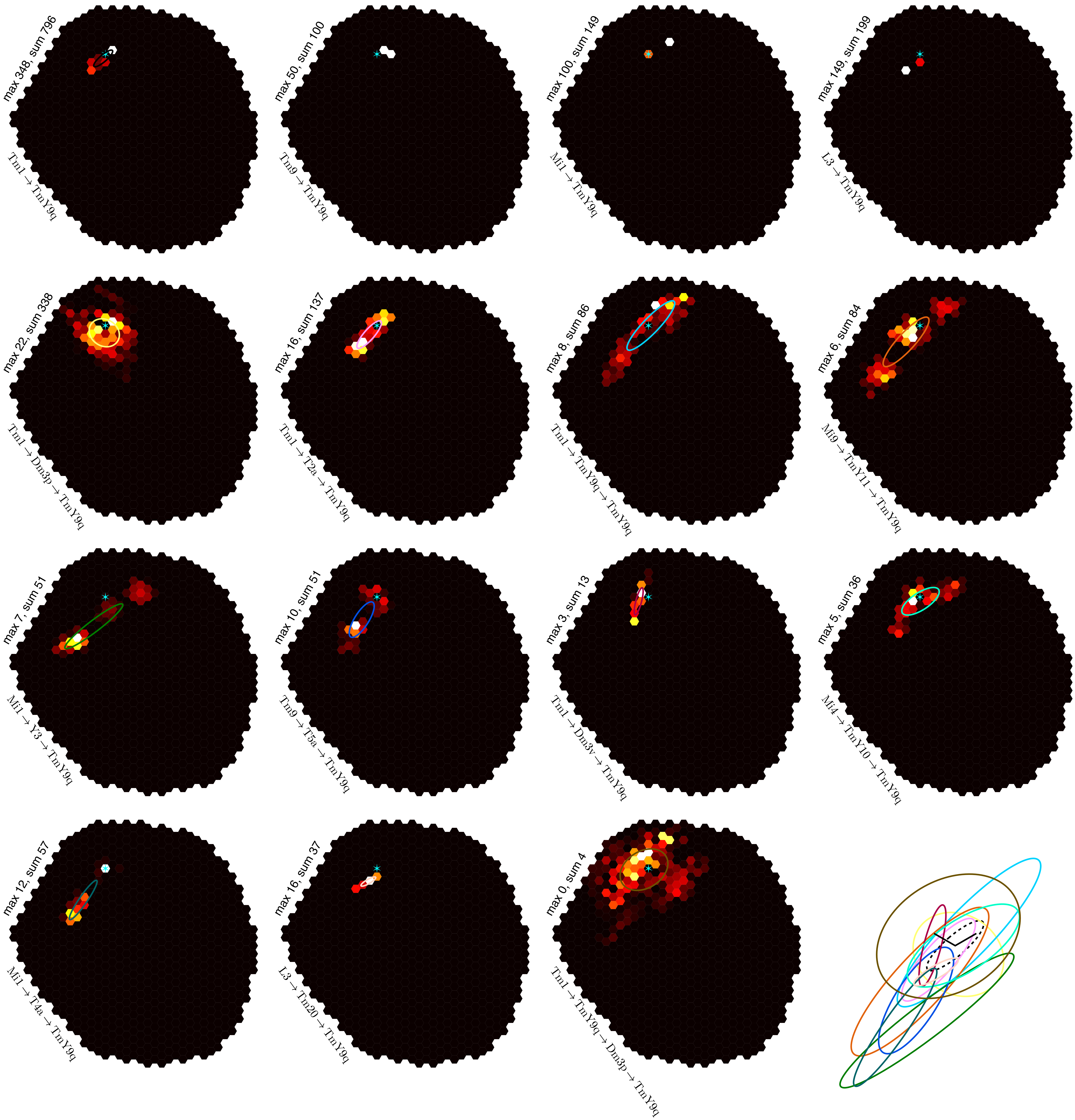
max 348, sum 796
Tm1 → TmY9q
max 50, sum 100
Tm9 → TmY9q
max 100, sum 149
Mi1 → TmY9q
max 149, sum 199
L3 → TmY9q
max 22, sum 338
Tm1 → Dm3p → TmY9q
max 16, sum 137
Tm1 → T2a → TmY9q
max 8, sum 86
Tm1 → TmY9q → TmY9q
max 6, sum 84
Mi9 → TmY11 → TmY9q
max 7, sum 51
Mi1 → Y3 → TmY9q
max 10, sum 51
Tm9 → T5a → TmY9q
max 3, sum 13
Tm1 → Dm3v → TmY9q
max 5, sum 36
Mi4 → TmY10 → TmY9q
max 12, sum 57
Mi1 → T4a → TmY9q
max 16, sum 37
L3 → Tm20 → TmY9q
max 0, sum 4
Tm1 → TmY9q → Dm3p → TmY9q
